# Assessment and validation of the CAESAR predictive model for bioconcentration factor (BCF) in fish

**DOI:** 10.1186/1752-153X-4-S1-S1

**Published:** 2010-07-29

**Authors:** Anna Lombardo, Alessandra Roncaglioni, Elena Boriani, Chiara Milan, Emilio Benfenati

**Affiliations:** 1 Laboratory of Chemistry and Environmental Toxicology, Istituto di Ricerche Farmacologiche Mario Negri, Milan, Italy

## Abstract

**Background:**

Bioconcentration factor (BCF) describes the behaviour of a chemical in terms of its likelihood of concentrating in organisms in the environment. It is a fundamental property in recent regulations, such as the European Community Regulation on chemicals and their safe use or the Globally Harmonized System for classification, labelling and packaging. These new regulations consider the possibility of reducing or waiving animal tests using alternative methods, such as *in silico *methods. This study assessed and validated the CAESAR predictive model for BCF in fish.

**Results:**

To validate the model, new experimental data were collected and used to create an external set, as a second validation set (a first validation exercise had been done just after model development). The performance of the model was compared with BCFBAF v3.00. For continuous values and for classification purposes the CAESAR BCF model gave better results than BCFBAF v3.00 for the chemicals in the applicability domain of the model. R^2 ^and Q^2 ^were good and accuracy in classification higher than 90%. Applying an offset of 0.5 to the compounds predicted with BCF close to the thresholds, the number of false negatives (the most dangerous errors) dropped considerably (less than 0.6% of chemicals).

**Conclusions:**

The CAESAR model for BCF is useful for regulatory purposes because it is robust, reliable and predictive. It is also fully transparent and documented and has a well-defined applicability domain, as required by REACH. The model is freely available on the CAESAR web site and easy to use. The reliability of the model reporting the six most similar compounds found in the CAESAR dataset, and their experimental and predicted values, can be evaluated.

## Background

Bioconcentration factor (BCF) is a fundamental property, describing the likelihood of a chemical concentrating in organisms, when the compound is present in the environment. It is required for regulatory purposes, for instance within the REACH (Registration, Evaluation, Authorisation and Restriction of Chemicals) [1] and Globally Harmonized System (GHS) [2] regulations. The first aims to ensure a high level of protection of human health and the environment as well as the free movement of substances, on their own, in preparations and in articles, while enhancing competitiveness and innovation. This regulation also promotes the development of alternative methods for assessing hazard of substances.

The GHS is intended to create a globally harmonized system of classification and labelling of chemicals to reduce the potential for adverse effects to people or the environment and to harmonize the existing classification and labelling systems. In Europe it is implemented through the Classification Labelling and Packaging (CLP) regulation [3], which is integrated into REACH (for classification and labelling). Under REACH, BCF data is required for all compounds produced or imported over 100 tonnes/y. BCF values are necessary for the classification and labelling, for the Persistent, Bioaccumulative and Toxic (PBT) evaluation and for chemical safety assessment. The first is obligatory for all chemicals produced or imported over one tonnes/y. The last two are usually required for substances produced or imported over ten tonnes/y. According to CLP, BCF data, with biodegradability, is necessary to assess whether a substance belongs to a chronic toxicity category. Table 1 summarises all the standard requirements for BCF.

**Table 1:** Summary of the standard requirements for BCF (Bioconcentration factor).

tonnage	REACH annexes	C&L (CLP)^1^	PBT^2 ^assessment	CSA^3^
*1 - 10 tonnes/y*		x (if available)		
*10 - 100 tonnes/y*		x (if available)	x	x
*> 100 tonnes/y*	x	x (if available)	x	x

^1 ^Classification and Labelling (Classification, Labelling and Packaging)

^2 ^Persistent, Bioaccumulative and Toxic

^3 ^Chemical Safety Assessment

The test is preferably done according to guidelines; REACH recommends fish as preferred species, and the corresponding OECD technical guideline is OECD 305 [4]. The test can use even hundreds of animals, taking more than a month, and costs several tens of thousands of Euros per chemical, depending on the country. This endpoint has been estimated to be one of those that will require more animal use (and more money, as a consequence) to comply with REACH [5,6].

REACH promotes the use of alternative methods to reduce the number of animal tests, and lists the *in silico *methods Quantitative Structure-Activity Relationship (QSAR), read-across and analogue identification. QSAR are mathematical models that seek a relationship between the chemical properties of a substance and its activity (e.g. toxicity, ability to bioaccumulate in organisms, carcinogenicity and so on). QSAR are also mentioned in other regulations, and indeed in the USA they have been applied for decades to assess chemical safety when experimental data are lack. A QSAR model (BCFBAF v3.0) has been developed to predict BCF and is present in the EPISuite v4.0, supported by US EPA (Environmental Protection Agency of United States).

The European Commission funded a project, CAESAR [8], to develop QSAR models taking account of the REACH requirements. CAESAR developed several models for five endpoints: BCF, mutagenicity, carcinogenicity, developmental toxicity and skin sensitisation. Then one model for each endpoint was chosen and included in the CAESAR web tool.

The CAESAR BCF model has been presented in a previous paper [9]. Here, we present the applet developed for using this BCF software, the validation of the CAESAR model with new data and its use in classification. Indeed, after CAESAR modelling activities had started, two data collections appeared, one from EURAS [10] and one from Canada [11]. The authors of both collections had carefully evaluated the data. Here, we discuss the overall results on the basis of the new validation and address the applicability domain of the model.

## Results

### The evaluation of the experimental data

The data, its quality and number are at the basis of any QSAR model. Good quality data is very important to obtain a good QSAR model. Data quality is even more important in the case of read-across, which relies on very few values. Data quality is anyway at the basis of any assessment, in particular for regulatory purposes. Sound assessment is necessarily based on sound data.

To properly assess the performance of a QSAR model it is important to know the specific variability of the endpoint of interest, as it will be implicitly transferred into the uncertainty of the QSAR model. A QSAR model cannot achieve predictions that are more accurate than the original data.

The variability of the BCF data reported in the literature is ± 0.75 log units [12]. The variability of the experimental data (calculated as the average of the range assumed by the values for each compound) in the Arnot *et al*. [11] database is 0.69 log units. Considering only experimental data for fish species suggested by the OECD (according to OECD guideline 305) and with an overall reliability score of 1 (the most reliable data), the variability drops to 0.48 log units. For the EURAS database, considered a gold standard database, the variability of the experimental BCF values is 0.45 log units, which decreases to 0.42 log units for the substances included in this study.

### LogP and its relationship with the BCF data

**Figure 1:**
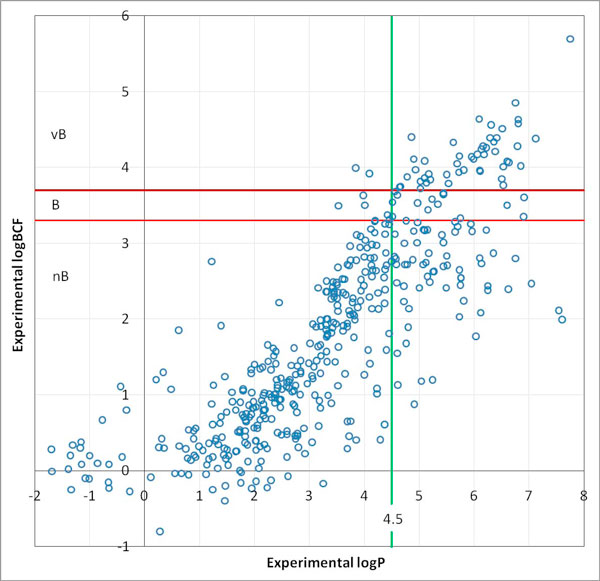
**Comparison of experimental values of logP and logBCF**. The two thresholds for BCF indicated in the REACH legislation are shown. The screening threshold proposed by ECHA for logP 4.5 is also reported.

LogP is the logarithm of the partition coefficient between octanol and water. It is considered very important to assess the bioaccumulation potential of a substance. Most models use logP to predict BCF (alone or together with other descriptors). The guidelines of the European Chemicals Agency (ECHA) for REACH suggest using logP for screening (if logP < 4.5, then the substance is non-bioaccumulative). Comparing the experimental values for logBCF and logP (see Figure 1), experimental logP alone cannot separate compounds that are bioaccumulative or not. Table 2 compares the results of the logP-based screening suggested by ECHA with experimental data. There are almost 2% of false negatives and the compounds with logP ≥ 4.5 are almost equally nB or B/vB (where nB means non-bioaccumulative, B bioaccumulative and vB very bioaccumulative). False negatives are compounds that are predicted as safe, without risky properties, but are in fact dangerous. Regulators want to avoid this situation. False positives are compounds that are predicted to be active, but are not.

**Table 2:** Confusion matrix for logBCF classification using logP experimental values.

		logP	
		
		< 4.5	≥ 4.5
**logBCF**	**nB^1^**	70.48%	14.32%
	**B^2^**	1.10%	3.08%
	**vB^3^**	0.44%	10.57%

^1 ^Non Bioaccumulative

^2 ^Bioaccumulative

^3 ^Very Bioaccumulative

The CAESAR model, like most of the QSAR models for BCF, used mainly log P as fundamental descriptor. So it is quite similar to models like BCFBAF v3.00 [7] and many others. We used a series of log P values calculated with four programs at pH 7, as explained in the past [9]. Table 3 reports the correlations between these calculated log P values and the experimental logBCF, for the chemicals used in the CAESAR model. These results do not mean that one program gives more reliable log P prediction; we simply explain the reasons for our selection in this specific case. When the model was developed, we also used logD as additional descriptor, calculating the partition coefficient in a series of acidic and basic pH, but the results were no better.

**Table 3:** Regression coefficient between logP calculated with different programs and BCF.

Descriptor	Chemical meaning	Source	Model	R	R^2^	F value
logP_ACD_	logP value calculated by ACD software	ACD software	log*BCF *= 0.305* logP_ACD_ +0.767	0.605	0.336	217.442
logP_Kowwin_	logP value calculated by Kowwin software	Kowwin software	log*BCF *= 0.357* logP_Kowwin_ +0.605	0.657	0.432	266.931
logP_MDL_	logP value from MDL descriptors	MDL descriptors	log*BCF *= 0.481* logP_MDL_ +0.290	0.737	0.543	448.043
MLOGP	Moriguchi octanol-water partition coeff. (logP)	Dragon software	log*BCF *= 0.555* MLOGP +0.117	0.746	0.556	471.748

As also appears from Table 3, the correlation between log P and BCF is not enough to support the use of this single parameter with a simple model. This is the same message as in Figure 1, where experimental values were used. Different factors are indeed involved in the BCF process and further components are necessary to simulate this better. Thus, seven other descriptors were identified using powerful information technology tools to screen a large number of potentially useful descriptors. Here we use the term descriptors in its broad sense, including molecular descriptors and fragments. Existing software, like the programs we used and describe in the experimental section, can calculate a large number of descriptors, considering the molecule as whole, or counting smaller molecular parts, like atoms or molecular groups. In this way, the model can be improved by extracting knowledge related to molecular descriptors, which boost its performance, taking account of other molecular features related to the property of interest. The CAESAR model includes two independent models, which run in parallel, and the results are combined in an integrated model [9].

### Validation of the CAESAR model

A major criticism of QSAR models is that they reflect the current list of chemicals used to build up the model, but they cannot predict the values for new compounds. For this reason, great care is needed in validating the QSAR model, using good statistical methods. There is still a debate in the QSAR community on the best ways to verify whether the model is predictive or not. Some authors prefer external validation, which is done with a set of compounds never used during the development of the model. This approach is questioned by others, who note that in some cases the number of compounds is too limited to use this approach without renouncing a significant proportion in order to represent the real situation; furthermore, external validation is related to the specific list of compounds, which can represent a bias. Thus, other methods are suggested, preferring internal validation.

**Figure 2:**
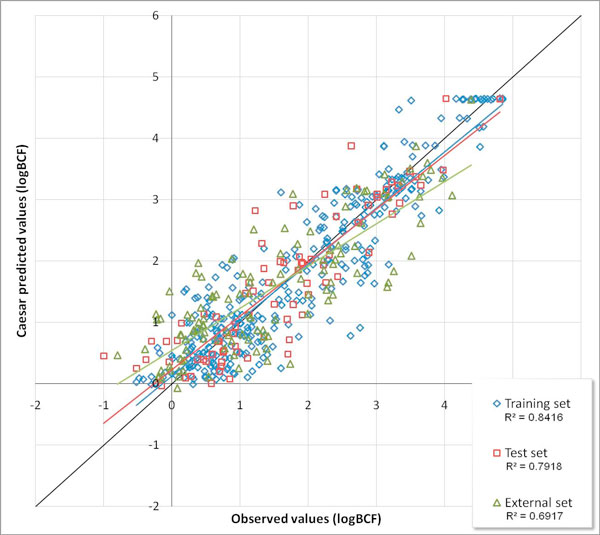
**CAESAR model performance**. Comparison of the experimental logBCF values and the predicted ones using the CAESAR model (chemicals within the applicability domain), for the training, validation and external sets.

We have presented the results of internal validation and the first set used for validation [9]. Figure 2 shows the results of the BCF models on the training set, on the first validation set and on the new, second validation set. The standard deviation error in prediction (SDEP) of the CAESAR BCF model is about 0.5 [9]. This is in agreement with the variability of the experimental data, and shows that on the average the expected errors of the *in silico *and experimental methods are similar. The following results consider only the compounds within the applicability domain of the CAESAR model (see the Experimental section). Thus, the number of compounds used for the evaluation as second validation set is 119, compared to 327 present in the training set. It means that the proportion is close to the 36% of the training set. The overall R^2 ^(the square correlation coefficient between predicted and experimental values) is 0.81, and the R^2 ^for the second validation set is 0.69. The SDEP is 0.57 for all the compounds, and 0.69 for the second validation set. To evaluate the performance of the model better we also considered the Q^2 ^(calculated according to [13]) of the entire dataset and of the external one, and the results were practically identical to R^2^, showing that the CAESAR model is predictive even if there is a reduction of the statistical characterisation.

**Figure 3:**
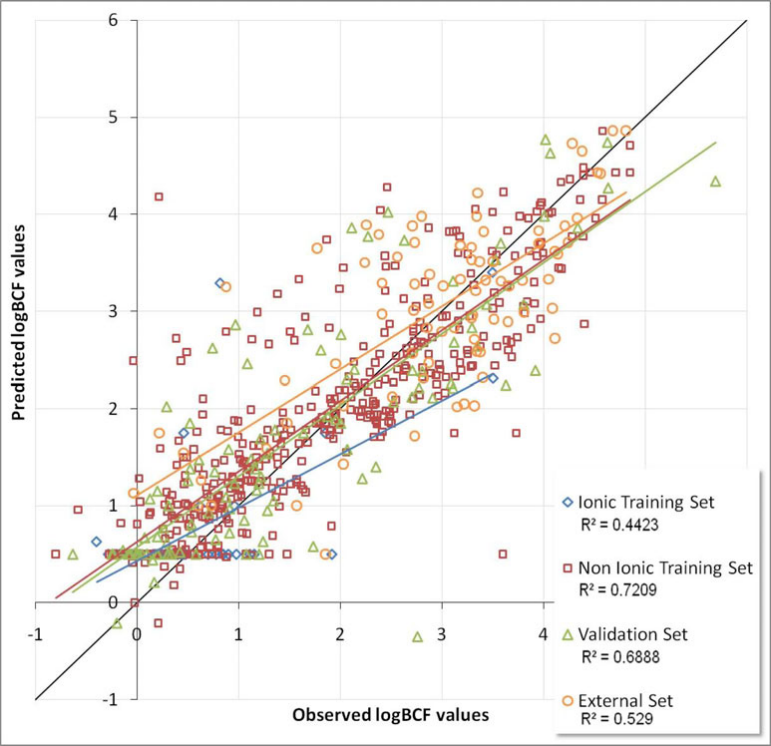
**BCFBAF v3.00 performance**. Comparison of the experimental logBCF values and the predicted ones using the BCFBAF v3.00 model for the ionic training, non-ionic training, validation and external sets.

Figure 3 shows the performance of BAFBCF v3.00 model reporting the results for the compounds used by the developers in their validation and training sets. BCFBAF v3.00 split chemicals in ionic and non-ionic, so we left this information. The developers did not use the compounds in the external during the model development. This external set is of 82 compounds and many of the compounds we took from Arnot were already present in the BAFBCF v3.00 training set. Compared to the 450 compounds in the BAFBCF v3.00 training set, the number of compounds (82) in this external validation set amount to 18%.

We also checked the performance, using the three splits (training, validation done by the developers, second validation with new compounds), and comparing the results for these splits according to CAESAR and BCFBAF v3.00. This meant the comparison was not biased by one splitting procedure, because all possibilities were assessed. Table 4 shows the results, indicating the R^2 ^and the SDEP. We excluded compounds that CAESAR labels as unreliable.

**Table 4:** R^2 ^and SDEP for CAESAR and BCFBAF v3.00 models.

*Set*	*CAESAR training*		*CAESAR test*		*CAESAR validation*	
***Model***	**CAESAR**	***BCFBAF v3.00***	**CAESAR**	***BCFBAF v3.00***	**CAESAR**	***BCFBAF v3.00***

***No. values***	327	*327*	81	*81*	119	*119*
***R***^2^	0.85	*0.80*	*0.83*	*0.77*	0.69	*0.61*
***SDEP***	0.53	*0.62*	0.51	*0.61*	0.70	*0.85*

***Set***	***BCFBAF training***		***BCFBAF validation***		***BCFBAF external***	

***Model***	**CAESAR**	***BCFBAF v3.00***	**CAESAR**	***BCFBAF v3.00***	**CAESAR**	***BCFBAF v3.00***

***No. values***	*383*	*383*	80	*80*	64	*64*
***R***^2^	0.79	*0.76*	0.78	*0.67*	0.79	*0.79*
***SDEP***	0.57	*0.64*	0.61	*0.75*	0.49	*0.81*

***Set***	***CAESAR validation ∩ BCFBAF v3.00 validation***		***CAESAR validation ∩ BCFBAF v3.00 external***		***total***	

***Model***	**CAESAR**	***BCFBAF v3.00***	**CAESAR**	***BCFBAF v3.00***	**CAESAR**	***BCFBAF v3.00***

***No. values***	22	*22*	7	*7*	527	*527*

***R***^2^	0.74	*0.68*	0.61	*0.19*	0.81	*0.75*

***SDEP***	0.64	*0.69*	0.72	*1.25*	0.57	*0.68*

R^2^, SDEP and number of compounds are reported for both models for the following sets of compounds: CAESAR (training, test and external), BCFBAF v3.00 (training, validation and external), compounds shared between CAESAR validation and BCFBAF v3.00 validation, compounds shared between CAESAR validation and BCFBAF v3.00 external and total compounds. Only the compounds in the applicability domain of CAESAR were analysed.

### Classification approaches for BCF

Using a quantitative model like CAESAR as a basis for classification approaches for BCF has the main advantage that its use remains flexible, not linked to a specific threshold, such as those indicated in specific legislations (for instance, a substance is considered bioaccumulative for REACH if the BCF value is greater than 2000, but for CLP the threshold is 500). Therefore, it can still be used if these limits are modified or updated over the years. In this section, we analyze the use of the CAESAR model in classification according to the REACH. Depending on the tonnage of the chemical to be put on the market, REACH specifics different ways to report the BCF characterisation. As already explained, for lower tonnage the information is only categorical, to define the chemical as bioaccumulative or not; however, at higher tonnage (> 100 tonnes/y) BCF has to be given as a continuous value to be used for risk evaluation.

Table 5 shows the results of the model (considering only compounds in the applicability domain), used for classification in three classes with the B and vB limits indicated in REACH: 3.3 in log units for B and 3.7 for vB. To take account of the uncertainty related to experimental and predicted values, an offset of 0.5 log units was applied to the compounds whose predicted BCF values fell near the B and vB thresholds. In other words, we applied a conservative criterion, reflecting the fact that the data are affected by a given uncertainty. In Table 5, we note that when used as a classifier the CAESAR model has clear advantages over the single criterion of the logP at 4.5 (see above) because: 1) it can predict three classes; 2) the accuracy of the prediction is much higher (always above 90% even on the second validation set, while accuracy for logP as from Table 2 is about 84%). Table 6 shows the confusion matrix using the CAESAR model as a classifier, with the 0.5 offset explained above. The percentage of false negatives decreases, but false positives increase. This solution is more conservative, as explained.

**Table 5:** Classification with the CAESAR model.

*Training set*		Observed logBCF			*First validation set*		Observed logBCF			*Second validation set*		Observed logBCF		
								
327 comp.		nB	B	vB	81 comp.		nB	B	vB	119 comp.		nB	B	vB
**Predicted logBCF**	**nB**	82.46 (270)	3.38 (11)	0.31 (1)	**Predicted logBCF**	**nB**	90.00 (72)	3.75 (3)	0.00 (0)	**Predicted logBCF**	**nB**	88.24 (105)	4.20 (5)	0.84 (1)
	**B**	1.54 (5)	2.15 (7)	0.92 (3)		**B**	0.00 (0)	1.25 (2)	1.25 (1)		**B**	0.84 (1)	1.68 (2)	2.52 (3)
	**vB**	0.62 (2)	1.23 (4)	7.38 (24)		**vB**	1.25 (1)	0.00 (0)	2.50 (2)		**vB**	0.00 (0)	0.84 (1)	0.84 (1)

Three sets are reported: training, first validation and second validation set. The percentage of the total of compounds predicted is given without considering those outside the applicability domain. In brackets, the number of compounds for each class. The total number of compounds is also reported.

**Table 6:** Classification with the CAESAR model adding a 0.5 log units offset.

*Training set*		Observed logBCF			*First validation set*		Observed logBCF			*Second validation set*		Observed logBCF		
								
327 comp.		nB	B	vB	81 comp.		nB	B	vB	119 comp.		nB	B	vB
**Predicted logBCF**	**nB**	73.70 (241)	0.31 (1)	0.00 (0)	**Predicted logBCF**	**nB**	77.78 (63)	0.00 (0)	0.00 (0)	**Predicted logBCF**	**nB**	81.51 (97)	1.68 (2)	0.00 (0)
	**B**	8.87 (29)	3.06 (10)	0.31 (1)		**B**	11.11 (9)	3.70 (3)	0.00 (0)		**B**	7.56 (9)	1.68 (2)	0.84 (1)
	**vB**	2.14 (7)	3.36 (11)	8.26 (27)		**vB**	1.23 (1)	2.47 (2)	3.70 (3)		**vB**	0.84 (1)	2.52 (3)	3.36 (4)

Three sets are reported: training, first validation and second validation set. The percentage of the total of compounds predicted is given without considering those that are outside the applicability domain. In brackets, the number of compounds for each class. The total number of compounds is also reported. To take account of the endpoint variability, the predicted values are modified adding an offset of 0.5 log units for the compounds near the B and vB thresholds.

**Figure 4:**
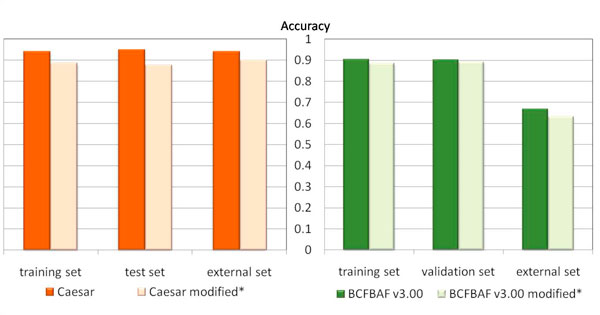
**CAESAR and BCFBAF v3.00 accuracy**. Comparison of the accuracy, using CAESAR and BCFBAF v3.00, for their three respective sets (training, validation and external). * Modified: using an offset of 0.5 for values close to the thresholds (see text).

The performance in classification of the CAESAR model (without and with the 0.5 correction) was compared with that of BCFBAF v3.00 (see Tables 7 and 8). Figure 4 shows the comparison of the accuracy of the models.

**Table 7:** Classification with the BCFBAF v3.00 model.

*Training set*		Observed logBCF			*Validation set*		Observed logBCF			*External set*		Observed logBCF		
								
450 comp.		nB	B	vB	103 comp.		nB	B	vB	82 comp.		nB	B	vB
**Predicted logBCF**	**nB**	82.00 (369)	3.33 (15)	2.00 (9)	**Predicted logBCF**	**nB**	81.55 (84)	2.91 (3)	1.94 (2)	**Predicted logBCF**	**nB**	39.02 (32)	12.20 (10)	3.66 (3)
	**B**	2.00 (9)	0.67 (3)	2.00 (9)		**B**	0.97 (1)	0.97 (1)	0.00 (0)		**B**	10.98 (9)	4.88 (4)	4.88 (4)
	**vB**	2.22 (10)	0.89 (4)	4.89 (22)		**vB**	3.88 (4)	0.97 (1)	6.80 (7)		**vB**	6.10 (5)	3.66 (3)	14.63 (12)

Three sets are reported for the compounds of the dataset: training, validation and external. The percentage of the total of compounds predicted is given without considering those outside the applicability domain. In brackets, the number of compounds for each class is reported. The total number of compounds is also reported.

**Table 8:** Classification with the BCFBAF v3.00 model adding a 0.5 log units offset.

*Training set*		Observed logBCF			*Validation set*		Observed logBCF			*External set*		Observed logBCF		
								
450 comp.		nB	B	vB	103 comp.		nB	B	vB	82 comp.		nB	B	vB
**Predicted logBCF**	**nB**	77.56 (349)	2.22 (10)	0.44 (2)	**Predicted logBCF**	**nB**	78.64 (81)	1.94 (2)	0.97 (1)	**Predicted logBCF**	**nB**	26.83 (22)	6.10 (5)	1.22 (1)
	**B**	4.44 (20)	1.11 (5)	1.56 (7)		**B**	2.91 (3)	0.097 (1)	0.97 (1)		**B**	12.20 (10)	6.10 (5)	2.44 (2)
	**vB**	4.22 (19)	1.56 (7)	6.89 (31)		**vB**	4.85 (5)	1.94 (2)	6.80 (7)		**vB**	17.07 (14)	8.54 (7)	19.51 (16)

Three sets are reported for the compounds of the dataset: training, validation and external. The percentage of the total of compounds predicted is given without considering those that are outside the applicability domain. In brackets, the number of compounds for each class. The total number of compounds is also reported. To take account of the endpoint variability, the predicted values are modified adding an offset of 0.5 log units for the compounds near the B and vB thresholds.

## Discussion

The BCF model developed within the CAESAR project proved to be predictive on the basis of the further validation done with a second validation set of 172 compounds. This shows the robustness, reliability and predictivity of the model. This model also proved valid on the basis of this demanding validation which is not generally done. Indeed, the second validation set is larger than the first, and its population is expected to be more heterogeneous. We verified that the first validation set was representative of the training set. Conversely, the new set of validation compounds included all compounds for which we found new data from the sources mentioned, and is thus probably more heterogeneous than the first validation set, which had the same data source.

Using the second validation set, the SDEP is still comparable with the experimental variability, which range from 0.75 to 0.42 for the sets of substances we used (see above). The limited increase of SDEP is partially due to this experimental variability, and partially to the model.

REACH requires a series of features for the QSAR models. Validity is a first and we did a further check to shows the model's validity better. Another criterion is that the model must have good documentation which is reliable for REACH. This is a further constraint and CAESAR is fully transparent and documented. All data used to build up the model, all structures, and the algorithm are given; the algorithm has been detailed in the scientific literature, including a description of the code [9], and the structures and data are publicly available through the web [8]. The model starts from experimental data obtained following an official protocol documented and suitable for REACH. All chemical structures have been checked within CAESAR by at least two partner laboratories, and a series of compounds have been eliminated, for errors or lack of sufficient detail in the structure or experimental protocol. This shows the very high quality evaluation of the input data. Furthermore, the output of the model has been designed for use with REACH, keeping in mind the thresholds given by this legislation. The model has been optimised to reduce the number of errors, particularly false negatives. This proves that the model is suitable for the output specifications, for classification and labelling and risk assessment, as required by REACH.

**Figure 5:**
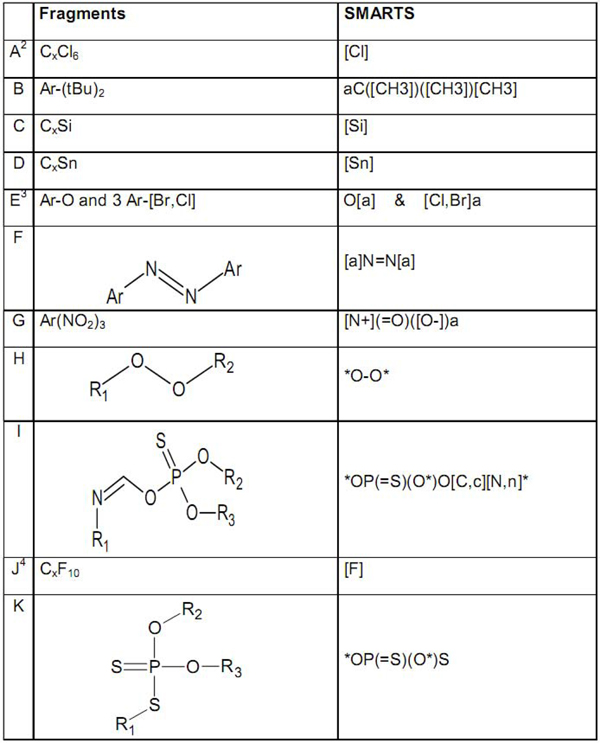
**Fragments related to large errors and corresponding SMARTS^1^**. ^1 ^SMARTS: SMiles ARbitrary Target Specification. ^2^A: using the [Cl] SMARTS all compounds with at least one chlorine atom are identified; to select only compounds with a minimum of six chlorine atoms the SMARTS recognizing program of the applet was used (it counts the number of fragments and then selects only the compounds with at least six). ^3^E: O[a] & [Cl,Br]a are two SMARTS to be used together to identify a fragment with an oxygen bound to an aromatic atom and at least three halogens (chlorine or bromine) bound to an aromatic (not necessary the same aromatic ring). ^4^J: for this SMARTS the explanation of the A point is valid.

The final requirement listed by REACH is regards the applicability domain of the model. This means assessing whether the model, even if good from a general point of view, is suitable to be applied to the specific chemical of interest. For BCF we developed a series of independent tools to assess this:

1. Chemical descriptor space. For instance, we excluded carbon disulfide because CAESAR reported the descriptors were out of the range.

2. Rules, codified into sub-structures that lead to greater uncertainty; they are identified by CAESAR using SMARTS (SMiles ARbitrary Target Specification) (see Figure 5). For instance, CAESAR identified a potential problem with a compound with Silicon.

3. Visualisation of similar substances. A tool was developed for this, showing the six compounds in the training set most similar to the predicted compound.

4. Measurement of the similarity. These six similar compounds are also related with a numerical score indicating the similarity with each compound of interest. The approach and algorithm are described in the experimental section. For instance, carbon disulfide had a poor similarity value, lower than 0.5.

5. We report the predicted value for each of these six similar compounds, compared with their experimental value, to give a direct appreciation of the potential errors. This further guides the user on the reliability. We give a couple of examples of the information sheets produced by CAESAR in the Supplementary Information.

Thus, we developed new tools for applicability domains, offering users information to assess whether a prediction is reliable for a certain compound. This battery of approaches for the applicability domain is innovative and complex. It uses not only *a priori *tools, based on chemometric measurements, as other methods do but we have added rules *a posteriori*, based on our results. Thus, these give a further evaluation, not only theoretical on the basis of chemical descriptors and fragments, but also on the basis of the output values and the observed errors.

These tools to identify pitfalls may help to explain why CAESAR performs better than BCFBAF v3.00. This latter identified only one substance potentially outside the applicability domain. Our approach gave much larger number of compounds improving the results on the remaining compounds. Figure 4 shows the performance of CAESAR using all possible splits of chemicals. The R^2 ^is always slightly higher than that of BCFBAF v3.00, and the SDEP, which shows the error, is always slightly lower. In one case the two models perform at the same level.

The user should always check and carefully evaluate the information given by CAESAR on the applicability domain. If there is a warning (for the range of descriptors or for the presence of critical fragments), or if the similarity of the chemical is not satisfactory, or if there are errors in the prediction of similar compounds, these factors should all lead to the conclusion that the model is not reliable for the chemical under evaluation.

If these factors are excluded, we can expect the error to be of the same order of magnitude as the experimental error. Further concern may arise when the predicted value is close to the threshold.

## Conclusions

The CAESAR model on BCF provides user with new tools for the prediction of this parameter. The model is publicly available, and has been designed to be easily usable. Nevertheless, a series of quality criteria have been introduced, keeping in mind the specific requirements of the REACH legislation, such as scientific validity (with two independent sets of compounds for validation), and clear, transparent documentation. The model has been designed to be suitable for REACH, considering the thresholds and legislative uses. Finally, innovative tools for a transparent check of the applicability domain have been developed and made publicly available through the web.

## Experimental

### The data

All chemical structures collected [12] were processed as explained [9]. The selected substances were split into the training (80% of the substances) and the test (20% of the substances) sets of the model.

The present study includes new data [10,11], carefully checked by the authors. The EURAS [10] only reports reliable BCF data for the fish indicated in the OECD 305 guidelines [4]. Instead, for the Arnot *et al*. [11] it was necessary to extract the most reliable BCF data (overall score of 1; endpoint 2: BCF-total water concentration) for the fish recommended by OECD 305 guidelines (*Danio rerio, Pimephales promelas, Cyprinus carpio, Oryzias latipes, Poecilia reticulata, Lepomis macrochirus, Oncorhynchus mykiss *and *Gasterosteus aculeatus*). For both the datasets, we further checked all chemicals, verifying the chemical structure (searching and checking the SMILES code) using public database online (ChemIDplus [14], PubChem Compound [15], Biodegradation and Bioconcentration of the Existing Chemical Substances [16], EPA DSSTox Search Tool [17], IBM Chemical Search Alpha [18] and InChI Converter [19]). All the chemicals with too little information to find the structure, the inorganic compounds, the isomer mixture, the metal complexes and the data from experiments on mixtures of chemicals were eliminated. The salts were neutralised. The list of compounds excluded is available in Additional files 1 and 2. We obtained 172 new compounds (reported in Additional file 3).

The chemical and experimental data of the CAESAR model are available at the CAESAR web site [8].

### The model

Details of the model have been published [9]. Briefly, descriptors were calculated using Dragon [20] (Talete, Milano, Italy) and MDL [21]. The model combines results of two independent models, offering greater accuracy. The two models were developed using support vector machine (SVM). The program R [22] and Matlab [23] were used to build up the model.

### Model validation

Within CAESAR the models were validated by both internal and external validation. The external validation was done in the past [9] using about 20% of the original compounds available when we started modelling. Here, the model was tested using a new external set obtained by combining the EURAS and the Arnot datasets, excluding the compounds already included in the CAESAR dataset. For the comparison we used the results of predictions for the model developed by Meylan *et al*. [24,25] and implemented in the BCFBAF v3.00 included into EPI Suite v4.0 [7].

The SDEP was calculated according to:

where o_i _are the observed values, p_i _the predicted values and n the number of values.

### Classification approach

The 635 compounds that form the complete dataset used for this work were split into training (370 compounds), first validation (93) and external sets (used as the second validation set of 172 compounds) of the CAESAR model. Because some of them are not in the applicability domain of the model, the three sets were reduced to 327, 81 and 119 compounds respectively. The percentage of the total compounds predicted is given without considering those that are outside the applicability domain. To get more conservative results, all the compounds near the two thresholds for B and vB compounds were raised 0.5 log units. To do this compounds between 2.8 and 3.3 were predicted as 3.31 and compounds between 3.31 and 3.7 as 3.71. Table 6 shows this modification.

Similarly, we split the 635 compounds into a training (as indicated by BCFBAF v3.00), test and external (never used by BCFBAF v3.00) sets. The results were analysed, making the confusion matrix reported in Table 7. In this case only one compound was outside the applicability domain of the model (defined from the molecular weight and logP), but it is well predicted, so it was not eliminated.

### Applicability domain

To evaluate the applicability domain we used three approaches. Additional 4 shows an example of CAESAR prediction for two compounds: octamethylcyclotetrasiloxane and carbon disulfide.

#### First approach: chemical descriptor space

The values for the training set of the eight descriptors in the combined model were used to define their ranges of validity. The CAESAR software gives a warning in this case.

#### Second approach: rules

A series of fragments, representing the compounds with greater uncertainty, were manually identified by searching among the structures with highest error (greater than 1 log unit) or misclassified (predicted nB when they are vB, or vice versa). These chemical features have been implemented in our model using short strings called SMARTS to define fragments. In addition, in this case the system gives the user a warning. SMARTS allows to specify substructures that are straightforward extensions of SMILES. Thus, flexible and efficient substructure-search specifications can be made in a way that is meaningful to chemists.

Two free programs have been used to do that: MarvinSketch [26] and Daylight Depict SMARTS Match [27]. The first is an advanced, Java-based chemical editor for drawing chemical structures, queries and reactions. We used it to draw 11 SMARTS fragments. To check the match between SMARTS and the actual sub-structure of interest, the Daylight Depict SMARTS Match was used, a web application based on Java code [28]. In this program the structure, depicted by a SMILES, is checked to find the fragment represented by the SMARTS.

The list of the SMARTS used in the model is reported in Figure 5.

#### Third approach: similarity tool

On the basis of several Dragon descriptors encoding different bi-dimensional characteristics of the molecules, a similarity index was developed to retrieve similar compounds from the CAESAR dataset, directly linked to the CAESAR models. More details of these tools are given in the paper on developmental toxicity [29], in this issue.

### Implementation in Java (the applet)

To facilitate the user, the model developed in other languages was implemented in Java. The software involves a dialogue between the server and the user, through the web. All calculations are done on the server. The user needs Java 1.6, which can be freely downloaded at the Sun Microsystem site [28]. For further description of the applet see [29].

### LogP - logBCF relationship

For this comparison we used only experimental logP values obtained from the Arnot database and the internal database of KOWWIN v1.67 (included into EPISuite v4.0 [7]). The experimental BCF values used for the comparison were obtained from two sources [9,10]. When two different logP or BCF values were reported, we used the average. In total, 454 compounds (on the 635 available) had an experimental logP value and were tested.

## Competing interests

The authors declare that they have no competing interests.

## Authors' contributions

Lombardo worked on DB development, performed the EPISuite v4.0 and CAESAR predictions, worked on validation of the model and manuscript drafting. Roncaglioni worked on the conceptual scheme of the applet, on the applicability domain and manuscript drafting. Boriani worked with DB curation and rules identification. Milan worked to develop the SMARTS. Benfenati worked on identification of rules and manuscript drafting. All authors read and approved the final manuscript.

## Supplementary Material

Additional file 1 **Compounds eliminated from the EURAS database **[10]**and the reasons for exclusion**. The CAS number and the reasons for exclusion are reported. Click here for file

Additional file 2 **Compounds eliminated from the Arnot database **[11]**and the reasons for exclusion**. The CAS number and the reasons for exclusion are reported. Click here for file

Additional file 3 **External dataset**. It reports the identification number, the CAS number, the chemical name, the SMILES, the source database (A for the Arnot database [11], E for the EURAS [10]), the experimental logP (the highest value of those reported in the Arnot database [11] and in the EPI [7] one), the mean of the experimental BCF and CAESAR hybrid model prediction. Click here for file

Additional file 4 **Two examples of the result sheets provided by CAESAR with indications of low reliability (chemicals outside the applicability domain)**. Click here for file
