# Supplementary material for: Assessment and validation of the CAESAR predictive model for bioconcentration factor (BCF) in fish
Source: Chem Cent J. 2010 Jul 29;4(Suppl 1):S1. doi: 10.1186/1752-153X-4-S1-S1 (PMC2913328; doi:10.1186/1752-153X-4-S1-S1)
Supplement: Additional file 4 — Two examples of the result sheets provided by CAESAR with indications of low reliability (chemicals outside the applicability domain). [file 1752-153X-4-S1-S1-S4.pdf]

CAESAR QSAR model for bioconcentration factor (BCF) in fish - version 1.0.0.11

The use of CAESAR models is regulated as in the license description available at the website.

More information on the CAESAR BCF model is available at the website.

http://www.caesar-project.eu

CAESAR QSAR model for bioconcentration factor (BCF) in fish

Prediction for the compound no. 1: C[Si]1(C)O[Si](C)(C)O[Si](C)(C)O[Si](C)(C)O1

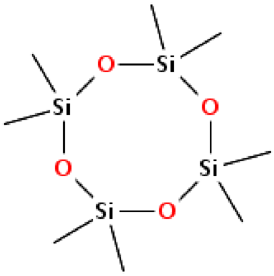	BCF value: 3 (L/Kg) whole body weight<br>Log BCF value: 0.47<br>Remarks for the prediction:<br>Presence of chemical features in the compound (Si atom in the molecule) that might be associated with a lower reliability of the predicted value.

The following chemicals similar to the query compound have been identified in the CAESAR database:

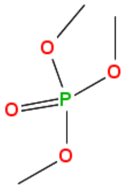	Dataset id: 406<br>SMILES: O=P(OC)(OC)OC<br>Similarity: 0.412<br><br>Experimental Log BCF: -0.52<br>Predicted Log BCF: 0.02
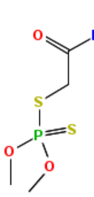	Dataset id: 464<br>SMILES: S=P(SCC(=O)NC)(OC)OC<br>Similarity: 0.404<br><br>Experimental Log BCF: -0.26<br>Predicted Log BCF: 0.09
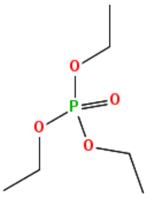	Dataset id: 404<br>SMILES: O=P(OCC)(OCC)OCC<br>Similarity: 0.386<br><br>Experimental Log BCF: -0.21<br>Predicted Log BCF: 0.58
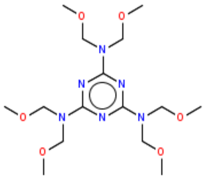	Dataset id: 442<br>SMILES: COCN(c1nc(nc(n1)N(COC)COC)N(COC)COC)COC<br>Similarity: 0.374<br><br>Experimental Log BCF: 0.28<br>Predicted Log BCF: 0.12
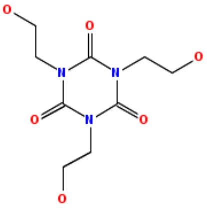	Dataset id: 444<br>SMILES: O=C1N(C(=O)N(C(=O)N1CCO)CCO)CCO<br>Similarity: 0.374<br><br>Experimental Log BCF: 0.20<br>Predicted Log BCF: 0.25
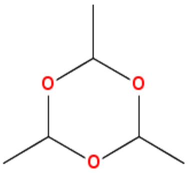	Dataset id: 462<br>SMILES: CC1OC(OC(O1)C)C<br>Similarity: 0.372<br><br>Experimental Log BCF: -0.52<br>Predicted Log BCF: 0.25

CAESAR QSAR model for bioconcentration factor (BCF) in fish

Prediction for the compound no. 2: S=C=S

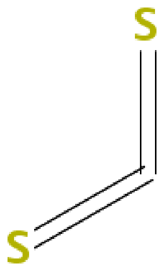	BCF value: 2 (L/Kg) whole body weight<br>Log BCF value: 0.24<br>Remarks for the prediction:<br>Descriptors for this compound have values outside the descriptor range for the compounds of the training set.

The following chemicals similar to the query compound have been identified in the CAESAR database:

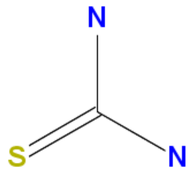	Dataset id: 399<br>SMILES: NC(=S)N<br>Similarity: 0.348<br><br>Experimental Log BCF: 0.30<br>Predicted Log BCF: 0.46
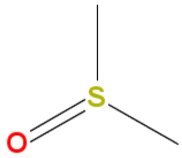	Dataset id: 398<br>SMILES: CS(=O)C<br>Similarity: 0.297<br><br>Experimental Log BCF: 0.60<br>Predicted Log BCF: 0.12
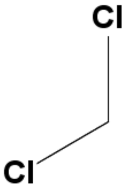	Dataset id: 240<br>SMILES: ClCCl<br>Similarity: 0.29<br><br>Experimental Log BCF: 1.37<br>Predicted Log BCF: 0.57
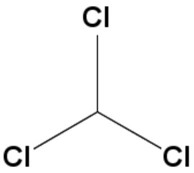	Dataset id: 241<br>SMILES: ClC(Cl)Cl<br>Similarity: 0.286<br><br>Experimental Log BCF: 0.93<br>Predicted Log BCF: 0.61
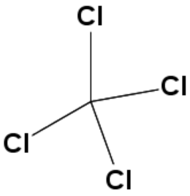	Dataset id: 242<br>SMILES: ClC(Cl)(Cl)Cl<br>Similarity: 0.283<br><br>Experimental Log BCF: 0.87<br>Predicted Log BCF: 0.61
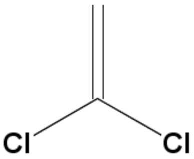	Dataset id: 259<br>SMILES: C=C(Cl)Cl<br>Similarity: 0.279<br><br>Experimental Log BCF: 0.95<br>Predicted Log BCF: 0.51
